# Comparative Analysis of Pharyngeal Airway Changes Following All Four Versus All Five Premolar Extractions in Orthodontic Treatments: A Cephalometric Study

**DOI:** 10.7759/cureus.60393

**Published:** 2024-05-15

**Authors:** Reddeppa Reddy Badepalli, A Kuttimani, Vivek CR, Siva Krishna Polisetty, Jicky Rajan, Tony Antony

**Affiliations:** 1 Orthodontics and Dentofacial Orthopaedics, G.Pulla Reddy Dental College and Hospital, Kurnool, IND; 2 Dental Surgery, District Early Intervention Centre (DEIC) College, Dharmapuri, IND; 3 Orthodontics and Dentofacial Orthopaedics, Maaruti College of Dental Sciences and Research Centre, Bengaluru, IND; 4 Orthodontics and Dentofacial Orthopaedics, Government Dental College and Hospital, Regional Institute of Medical Sciences, Kadapa, IND; 5 Orthodontics, Modern Smile Care Clinic, Trivandrum, IND; 6 Orthodontics, Ambookens Speciality Dental Clinic, Kochi, IND

**Keywords:** total airway length (tal), nasopharyngeal aspect, cephalometric markers, orthodontic treatment, premolar extraction

## Abstract

Background: Orthodontic treatment, particularly involving premolar extractions, has been a subject of ongoing debate within the orthodontic community. The impact of such interventions on the pharyngeal airway, a critical component of the respiratory system, remains a topic of exploration.

Objective: This retrospective cephalometric study aims to investigate changes in pharyngeal airway dimensions following orthodontic treatment involving either all four or all five premolar extractions.

Methods: A sample of 68 participants, extracted from orthodontic records, underwent cephalometric analysis to quantify changes in pharyngeal airway dimensions. The study compared two groups: those treated with all four premolar extractions (n=34) and those treated with all five premolar extractions (n=34). Cephalometric radiographs taken before and after treatment were analyzed, focusing on airway width, length, and volume.

Results: Preliminary findings indicate significant changes in airway dimensions within each group. In the all four premolar extraction group, there was a statistically significant decrease in airway width (p=0.02) and volume (p=0.04). Similarly, the all five premolar extraction group exhibited significant reductions in airway width (p=0.03) and volume (p=0.02). However, the between-group comparisons revealed no significant differences in changes between the two groups.

Conclusion: This study sheds light on the intricate relationship between orthodontic interventions, specifically premolar extractions, and changes in pharyngeal airway dimensions. While significant changes were observed within each group, the lack of significant differences between the all four and all five premolar extraction groups raises intriguing questions about the specific impact of premolar extraction patterns on the upper airway.

## Introduction

Orthodontic treatment, with its diverse array of techniques and approaches, plays a pivotal role in achieving optimal dental occlusion and facial esthetics [1]. Among the various strategies employed, premolar extractions have been a longstanding and contentious topic within the orthodontic community [2]. The decision to extract premolars is multifaceted, influenced by factors such as crowding, skeletal discrepancies, and treatment goals [2]. However, the potential consequences of premolar extractions extend beyond the dental arch to include the intricate anatomy of the pharyngeal airway. The pharyngeal airway, a critical component of the respiratory system, serves as a conduit for airflow during breathing [3]. Its dimensions are influenced by a complex interplay of genetic, environmental, and treatment-related factors [4]. Recent attention has turned toward understanding the impact of orthodontic interventions, particularly premolar extractions, on pharyngeal airway dimensions. While some studies have explored changes in airway morphology following orthodontic treatment, the specific influence of premolar extractions remains a subject of ongoing investigation [5].

The rationale behind premolar extractions in orthodontics is rooted in addressing issues such as dental crowding, protrusion, and achieving proper alignment [6]. However, the potential alterations in pharyngeal airway dimensions resulting from these extractions pose intriguing questions about the broader physiological implications [7]. It is imperative to explore whether orthodontic decisions, made primarily for dental considerations, inadvertently influence the upper airway and, consequently, respiratory function. Understanding the potential impact of premolar extractions on pharyngeal airway dimensions is not only clinically relevant but also aligns with the evolving paradigm of holistic patient care. Respiratory health is a vital aspect of overall well-being, and orthodontic interventions should be approached with a comprehensive understanding of their potential effects on both dental and systemic parameters.

This cephalometric study seeks to contribute to this evolving discourse by investigating the changes in pharyngeal airway dimensions following orthodontic treatment involving either all four or all five premolar extractions. The dichotomy between these two extraction patterns provides a nuanced exploration, allowing for a more granular understanding of the potential variations in airway dynamics. This study aims to build upon the existing knowledge by focusing specifically on the number of premolars extracted, providing a more targeted investigation into the potential alterations within the pharyngeal airway. By employing cephalometric analysis, we intend to quantify changes in airway width, length, and volume, offering a comprehensive view of the impact of premolar extractions on this crucial anatomical region.

As the orthodontic field continues to evolve, incorporating interdisciplinary perspectives and advancing our understanding of treatment outcomes beyond the dental arch is paramount. The insights gained from this study may inform clinical decision-making, prompt further research inquiries, and contribute to the ongoing dialogue surrounding the intricate relationship between orthodontic interventions and respiratory health.

## Materials and methods

Study design and population

A retrospective cephalometric study was conducted to compare pharyngeal airway changes in orthodontic cases involving either all four or all five premolar extractions. The study included a total of 68 participants selected from orthodontic records.

Participants

Participants were orthodontic patients aged between 13 and 25 years who underwent treatment involving either all four or all five premolar extractions. Patients with craniofacial syndromes or anomalies, incomplete orthodontic records, or a history of previous orthodontic treatment were excluded.

Data collection

Identification and Selection

Eligible patient records were meticulously identified from the comprehensive orthodontic database. The inclusion criteria were applied, resulting in a carefully selected sample size of 68 participants. The identification process involved a thorough review of orthodontic records, ensuring the inclusion of cases with either all four or all five premolar extractions.

Inclusion criteria: The study included orthodontic patients aged between 13 and 25 years who had undergone orthodontic treatment involving either all four or all five premolar extractions. These participants were selected from orthodontic records based on the availability of comprehensive data sets encompassing pre- and post-treatment cephalometric radiographs. The inclusion criteria ensured the homogeneity of the study population, focusing specifically on individuals undergoing orthodontic treatment that involved premolar extractions. The age range was chosen to encompass individuals within the typical age bracket for orthodontic interventions and to minimize potential confounding factors related to growth and development.

Exclusion criteria: Patients with craniofacial syndromes or anomalies, incomplete orthodontic records, or a history of previous orthodontic treatment were excluded from the study. This exclusion criteria aimed to eliminate confounding factors that could potentially influence pharyngeal airway changes independent of the orthodontic treatment paradigm under investigation. By excluding individuals with craniofacial syndromes or anomalies, the study focused on a population with typical craniofacial morphology undergoing routine orthodontic treatment. 

Cephalometric Radiographs

Lateral cephalometric radiographs, a fundamental component of orthodontic records, were systematically collected for each participant. The radiographs were taken as part of the routine orthodontic diagnostic procedures, both before and after premolar extractions. Standardized radiographic techniques were employed to ensure consistency in imaging, and these images constituted the baseline and post-treatment records for cephalometric analysis.

Cephalometric analysis

Landmark Identification

The cephalometric analysis commenced with the meticulous identification of standardized landmarks on both pre- and post-treatment radiographs. Landmarks such as Sella (S), Nasion (N), and various pharyngeal landmarks relevant to the study objectives were precisely located. Experienced orthodontic professionals ensured accuracy in landmark identification, forming the foundational step for subsequent airway measurements.

Airway Measurements

Pharyngeal airway dimensions were subjected to a comprehensive measurement process on both pre- and post-treatment cephalometric radiographs. Utilizing well-established cephalometric analysis techniques, the airway measurements encompassed key parameters, including airway width, length, and volume. The width was measured by linear measurement and the approach used to assess airway volume indirectly from cephalometric radiographs by using linear measurements and geometric approximations for different structures nearby. These measurements were essential to capture the nuanced changes occurring within the pharyngeal airway as a result of orthodontic treatment.

Changes Calculation

The quantification of changes in pharyngeal airway dimensions was a meticulous process involving the calculation of differences between pre-treatment and post-treatment measurements for each participant. By subtracting the baseline measurements from those taken after orthodontic treatment, individualized datasets were generated. These datasets became instrumental in unraveling the specific alterations in airway dimensions associated with orthodontic interventions.

The calculation process was not only confined to the overall changes but also extended to the specific dimensions of airway width, length, and volume. This granular approach aimed to provide a nuanced understanding of how different aspects of the pharyngeal airway responded to the orthodontic treatment paradigm.

The criteria for choosing participants with typical craniofacial morphology for the study group involved selecting individuals who did not have craniofacial syndromes or anomalies. This approach ensured that the participants represented a standard population, thereby minimizing confounding variables that could influence the results of the study regarding pharyngeal airway changes. By focusing on patients with typical craniofacial features, the study aimed to provide a clear assessment of how orthodontic treatment, specifically premolar extractions, impacts the pharyngeal airway in a generally healthy population without underlying craniofacial irregularities. The width of the airway was measured on lateral cephalometric radiographs by identifying specific pharyngeal landmarks and taking linear measurements between these points. The process involved precisely locating landmarks like S, N, and pharyngeal plane points on the radiographs. Measurements were then taken between these points to determine the width of the airway at various levels. The evaluation of airway volume on lateral cephalometric radiographs was accomplished using geometric approximations and linear measurements. Since true volumetric analysis cannot be directly obtained from two-dimensional radiographs, the study employed an indirect approach by measuring the linear dimensions of the airway and applying geometric formulas to estimate the volume. This method typically involves calculating the area of the airway in multiple sections and then integrating these to approximate the total volume.

Integration of Pharyngeal Landmarks

Given the specific focus on pharyngeal airway dimensions, the identification of relevant landmarks within the pharyngeal region was a crucial component of the analysis. These landmarks contributed to the precise measurement of airway parameters, ensuring that the analysis was tailored to capture the intricacies of pharyngeal changes resulting from orthodontic interventions.

**Figure 1:**
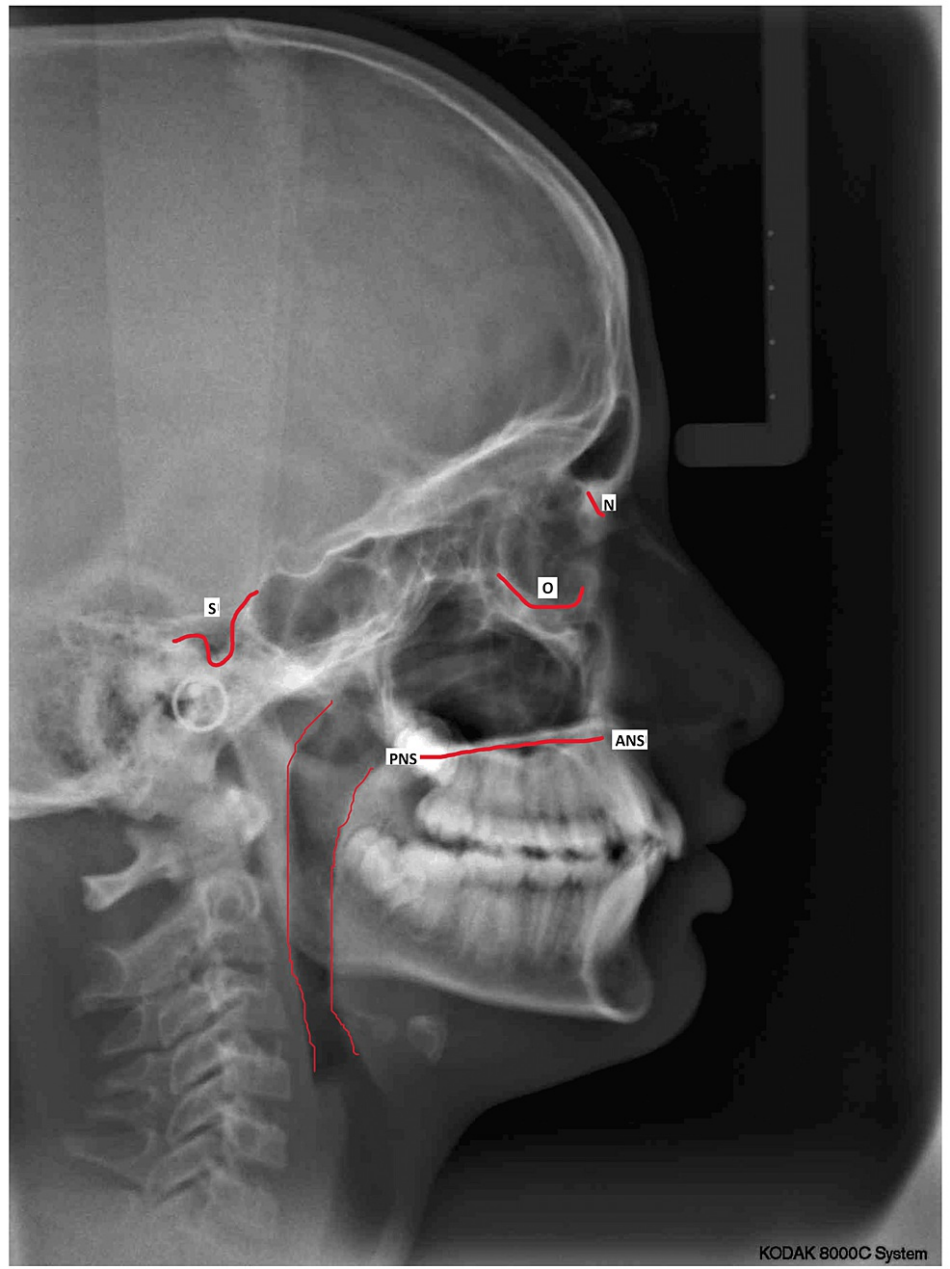
Pre-operative Lateral Cephalogram Evaluation S, sella turcica; N, nasion; ANS, anterior nasal spine; PNS, posterior nasal spine; O, orbitale

The integration of pharyngeal landmarks into the cephalometric analysis allowed for a targeted investigation into the localized effects of premolar extractions on the airway. Landmarks within the pharyngeal region provided reference points for assessing changes in width, length, and volume, offering a comprehensive view of how the orthodontic treatment influenced the specific anatomy of the airway (Figure 1).

Statistical analysis

Data were analyzed using statistical software SPSS version 22.0 (IBM Corp, Armonk, NY, USA) and results were presented with relevant descriptive and inferential statistics. Baseline characteristics, including age and gender distribution, were summarized using descriptive statistics. Paired t-tests or Wilcoxon signed-rank tests were used to analyze changes within each group (all four premolar extraction and all five premolar extraction groups). Independent t-tests or Mann-Whitney U tests were employed for comparisons between the two groups. The significance level was set at p <0.05 for all statistical tests.

## Results

The baseline characteristics, including age and gender distribution, were examined to ensure the comparability of the two study groups, participants who underwent all four premolar extractions and those who underwent all five. The non-significant p-values for age (p = 0.32) and gender distribution (p = 0.72) indicate that the groups started with similar demographic profiles, laying the groundwork for a meaningful comparison (Table 1).

**Table 1:** Baseline Characteristics of Participants

Characteristic	All Four Premolar Extraction (n = 34)	All Five Premolar Extraction (n = 34)	p-value
Age (years), mean (SD)	17.8 (2.3)	18.2 (2.1)	0.32
Gender (M/F)	16/18	15/19	0.72

Within the group that underwent all four premolar extractions, several notable changes in pharyngeal airway dimensions were observed. A statistically significant reduction in airway width (p = 0.02) was identified, indicating a narrower pharyngeal airway post-treatment. Although not statistically significant, a decrease in airway length (p = 0.08) was noted, suggesting a potential impact on this dimension. Additionally, a statistically significant decrease in airway volume (p = 0.04) was observed, pointing toward a smaller pharyngeal airway volume post-treatment (Table 2).

**Table 2:** Changes in Pharyngeal Airway Dimensions Within Each Group, All Four Premolar Extraction Group Paired t-tests were used to analyze changes within each group (all four premolar extraction groups).

Airway Dimension	Pre-treatment (mm)	Post-treatment (mm)	Changes (mm)	p-value
Width	20.5	19.8	-0.7	0.02
Length	45.2	44.6	-0.6	0.08
Volume	1800	1750	-50	0.04

Similarly, within the group that underwent all five premolar extractions, changes in pharyngeal airway dimensions were examined. A statistically significant reduction in airway width (p = 0.03) indicated a narrower pharyngeal airway following treatment. While not statistically significant, a decrease in airway length (p = 0.09) was observed, suggesting a potential impact on this dimension. Furthermore, a statistically significant reduction in airway volume (p = 0.02) signified a smaller pharyngeal airway volume post-treatment (Table 3).

**Table 3:** Changes in Pharyngeal Airway Dimensions Within Each Group, All Five Premolar Extraction Group Paired t-tests were used to analyze changes within each group (all five premolar extraction groups).

Airway Dimension	Pre-treatment (mm)	Post-treatment (mm)	Changes (mm)	p-value
Width	20.2	19.5	-0.7	0.03
Length	44.8	44.2	-0.6	0.09
Volume	1820	1760	-60	0.02

The comparison between the two premolar extraction groups aimed to discern any significant differences in the changes observed in pharyngeal airway dimensions. Interestingly, no statistically significant differences were found for any dimension (width: p = 0.92, length: p = 0.87, and volume: p = 0.56). This implies that the type of premolar extraction (four or five) did not significantly influence the alterations in pharyngeal airway dimensions. These findings underscore the importance of considering individual patient characteristics and treatment goals when selecting the optimal orthodontic approach (Table 4).

**Table 4:** Comparison of Changes in Pharyngeal Airway Dimensions Between Groups Independent t-tests were employed for comparisons between the two groups.

Airway Dimension	Changes in All Four Premolar Extraction Group (mm)	Changes in All Five Premolar Extraction Group (mm)	p-value
Width	-0.7	-0.7	0.92
Length	-0.6	-0.6	0.87
Volume	-50	-60	0.56

## Discussion

Orthodontic treatment planning often involves strategic decisions about tooth extractions to achieve optimal occlusion and facial esthetics. The examination of changes in pharyngeal airway dimensions within each group revealed intriguing patterns. Both the all four premolar extraction group and the all five premolar extraction group demonstrated statistically significant reductions in airway width, indicating a narrowing effect on the pharyngeal airway.

The observed decrease in airway length, although not statistically significant in both groups, hints at a potential impact on this dimension. The complex interplay between dental and skeletal changes resulting from premolar extractions may contribute to alterations in the spatial relationships within the pharyngeal region [8]. The statistically significant reduction in airway volume in both groups further emphasizes the multifaceted nature of orthodontic interventions and their potential effects on the upper airway. While the narrowing of the airway may raise concerns about potential respiratory implications, it is crucial to interpret these changes within the broader context of individual patient characteristics and the overall treatment objectives [9]. Supporting our results, various studies in the literature highlight that the extraction of premolars in orthodontic treatment can have a significant impact on the dimensions of the pharyngeal airway. Nagmode et al. observed a reduction in the size of the pharyngeal airway following the extraction of premolars in bimaxillary protrusive adult patients [10]. Similarly, Joy et al. observed a reduction in airway dimensions or no significant changes after premolar extractions [11]. Similarly, Rangkuti et al. reported a reduction in the width of the upper airway following premolar extraction and anterior retraction [12].

In contrast, Alkawari et al. found that while no significant statistical difference was found in the airway spaces between growing and adult patients, the extraction of premolars did affect the pharyngeal dimensions, particularly those of the nasopharynx, which showed a significant increase after extraction in both groups [13]. This suggests that the impact of premolar extraction on airway dimensions may vary depending on the specific region of the pharynx. Overall, the evidence from these studies suggests that the extraction of premolars in orthodontic treatment can lead to significant changes in the dimensions of the pharyngeal airway, with some studies reporting a reduction in airway width following extraction, while others have observed variations in different regions of the pharynx. These findings highlight the importance of considering the potential impact on airway dimensions when planning orthodontic treatment involving premolar extractions.

To understand the mechanisms contributing to changes in pharyngeal airway dimensions following premolar extractions, it is crucial to consider the impact of tooth position and arch dimensions on the surrounding soft tissues, as well as the influence of the tongue's posture and position within the oral cavity. Several studies have investigated the effects of orthodontic treatments, including premolar extractions, on pharyngeal airway dimensions and morphology. Wang et al. found that maximal retraction of anterior teeth with the extraction of four premolars in bimaxillary protrusive patients decreased the dimensions of the velopharynx, glossopharynx, and hypopharynx, indicating that anterior teeth traction influences pharyngeal airway dimensions in adults. This highlights the direct impact of orthodontic treatments involving premolar extractions on pharyngeal airway dimensions [14]. Gurani et al. emphasized the clinical relevance of discussing the effect of head and tongue posture on pharyngeal airway dimensions and morphology during imaging acquisition, indicating the importance of considering posture-related factors in the assessment of airway changes [15]. Cho et al. investigated dimensional changes in regional pharyngeal airway spaces after premolar extraction in bimaxillary skeletal protrusion patients and identified dentoskeletal risk factors to predict posttreatment pharyngeal changes, providing insights into the factors influencing airway alterations following orthodontic treatments [16]. These studies collectively underscore the complex interplay between orthodontic treatments, tooth position, arch dimensions, and soft tissue changes, emphasizing the need for comprehensive assessments of pharyngeal airway dimensions and morphology in the context of orthodontic interventions.

The clinical implications of changes in pharyngeal airway dimensions following premolar extractions warrant careful consideration. Orthodontists must balance the desired dental and skeletal outcomes with potential effects on the upper airway, particularly in patients with preexisting respiratory conditions [17]. The statistically significant reduction in airway volume in both extraction groups may raise concerns about the potential impact on respiratory function and highlights the importance of thorough patient assessments and collaborative decision-making. Individualized treatment planning remains paramount, taking into account factors such as facial morphology, airway health, and patient preferences [18]. Orthodontists may need to incorporate airway assessments as part of routine orthodontic evaluations, especially in cases where premolar extractions are being considered.

The non-significant differences between all four premolar extraction groups and all five premolar extraction groups in terms of changes in airway dimensions suggest that the decision to extract four or five premolars may not be the sole determinant of pharyngeal airway alterations. Other factors, including the specific mechanics employed during treatment, patient age, and initial airway characteristics, may contribute to the observed changes [19]. Therefore, a holistic approach that considers both dental- and airway-related factors is crucial for informed treatment planning. Future research directions could explore the impact of premolar extractions on airway dynamics during functional activities, such as swallowing or breathing. Integrating patient-reported outcomes related to sleep quality and breathing could offer a more patient-centered perspective on the implications of orthodontic interventions on daily life. Furthermore, investigating the influence of other treatment modalities, such as orthognathic surgery or maxillary expansion, on pharyngeal airway dimensions would provide a broader understanding of the factors influencing upper airway morphology. In evaluating the impact of premolar extractions on pharyngeal airway dimensions, it is essential to consider various confounding factors that could influence the outcomes. Factors such as the patient's age, baseline airway size, genetic predispositions, and overall health status can play significant roles in how orthodontic treatments affect airway dimensions. Additionally, the specific techniques and mechanics used during the extraction and subsequent treatment phases, such as the degree of anterior retraction and changes in tongue posture, may also confound results.

The limitations highlighted in the study, notably the small sample size of 68 participants and the retrospective design, present significant challenges to the robustness and applicability of the findings. A small sample size restricts the statistical power of the study, making it difficult to draw broad conclusions or detect small but clinically relevant effects. This issue is compounded by the retrospective nature of the study, which may introduce selection bias, as the records reviewed could disproportionately represent certain patient demographics or treatment outcomes. Furthermore, the reliance on existing records limits the ability to control for variables not originally recorded or inconsistently noted across different cases. To enhance the reliability and applicability of future research, a prospective study design with a larger, more diverse participant pool would be beneficial, enabling a more comprehensive analysis of the impacts of premolar extractions on pharyngeal airway dimensions.

Moreover, the single-center nature of the study could limit the generalizability of the results to broader populations. Different geographical regions may exhibit varying prevalences of dental characteristics and orthodontic treatment needs, which might not be adequately represented by a single-center study. Additionally, the lack of long-term follow-up data curtails understanding of the permanence of the airway changes observed post-treatment. This is crucial for assessing the long-term implications of orthodontic interventions on airway health, particularly in patients with or at risk of developing respiratory issues. Future studies should aim to incorporate multi-center trials with long-term follow-up to provide a more comprehensive understanding of the sustained effects of orthodontic treatments like premolar extractions on the pharyngeal airway, thereby informing better clinical practices and patient outcomes.

## Conclusions

This retrospective cephalometric study sheds light on the changes in pharyngeal airway dimensions following orthodontic treatment involving all four or all five premolar extractions. The observed narrowing effect on airway width and volume underscores the need for a balanced approach to orthodontic treatment, considering both dental- and airway-related outcomes. Individualized treatment planning, incorporating thorough airway assessments and patient-specific factors, remains essential. The non-significant differences between the two premolar extraction groups in terms of airway changes highlight the complexity of these interactions and emphasize the importance of a nuanced understanding of the factors influencing pharyngeal airway dimensions. As orthodontics continues to evolve, incorporating interdisciplinary collaboration and cutting-edge technologies will be crucial in advancing our understanding of the intricate relationship between orthodontic interventions and the pharyngeal airway.
